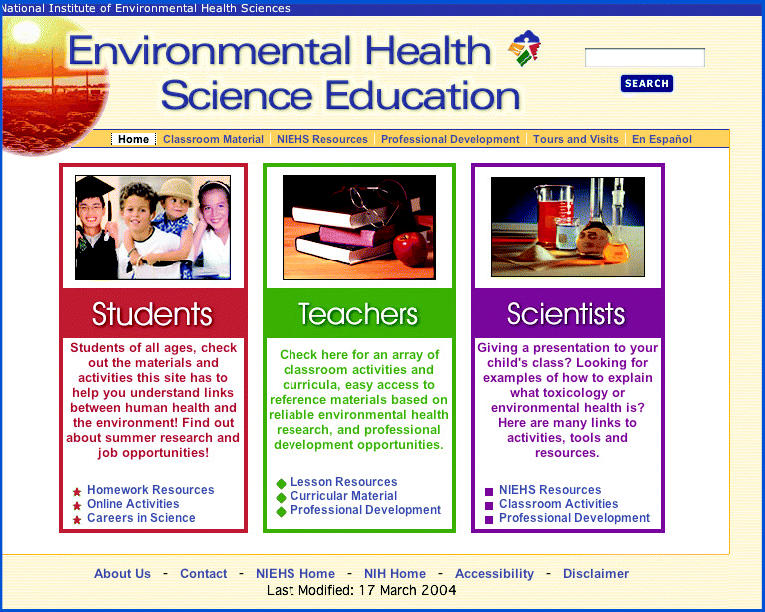# EHPnet: NIEHS Environmental Health Science Education

**Published:** 2004-10

**Authors:** Erin E. Dooley

Although the NIEHS is known primarily as a research institution, the institute is also charged with making its findings openly accessible to the public. As one of its efforts in this area, the NIEHS has developed its Environmental Health Science Education website to provide teaching materials for students and educators on topics including asthma, carcinogens, herbal medicines, nanotechnology, obesity, and risk assessment. To aid in the ease of finding materials, the site’s homepage, located at **http://www.niehs.nih.gov/science-education/home.htm**, has three portals, one for each of the three audiences—students, teachers, and scientists—that may be accessing the site.

The Students portion contains a library of homework resources, sorted by type of publication: factsheet, pamphlet, news article, or video. The page shows the ages for which each resource is intended. Within the Online Activities subsection are links to games, puzzles, tips for healthy living, environment-related coloring books, and storybooks. Most of these activities are intended for elementary or middle school students, although there is one tool, Project Greenskate, that has been developed for high school students. The Students portion also contains information on summer employment and training opportunities for high school and college students.

In the Teachers portion of the site, educators can find curricular materials, sorted by keywords, that include a real-time air quality activity, guides to performing scientific techniques such as the Ames assay, classroom role-playing scenarios such as the Hydroville Challenge project, and staff development units. Also within the curricular materials section are links to PDF presentations, web-based activities, and videos. Teachers can access lesson resources such as pamphlets and other print materials. Under the Professional Development subsection of the Teachers portion are links to the NIEHS Summers of Discovery program and other teacher training programs around the country.

The Scientists portion of the site, meanwhile, offers resources that environmental health professionals can use in making classroom presentations on subjects related to their work. The resources are annotated with the subject, format, and intended audience. Also within this section is a Professional Opportunities page, which links to *EHP*’s online Career Opportunities and Fellowships listings, as well as training, fellowship, and career development opportunities through the NIEHS Division of Extramural Research and Training.

All of the materials on the website are fully searchable from the homepage. There is also a menu bar across the top of the homepage to quickly access the most often requested pages. Visitors can find information on how to schedule tours to the NIEHS campus in Research Triangle Park, North Carolina, and how to request classroom visits by NIEHS scientists. A number of the resources available on the site are also available in Spanish. These can be accessed through a link on the top menu bar.

## Figures and Tables

**Figure:**